# Use of serum KL-6 level for detecting patients with restrictive allograft syndrome after lung transplantation

**DOI:** 10.1371/journal.pone.0226488

**Published:** 2020-01-13

**Authors:** Cristina Berastegui, Susana Gómez-Ollés, Alberto Mendoza-Valderrey, Thais Pereira-Veiga, Mario Culebras, Victor Monforte, Berta Saez, Manuel López-Meseguer, Helena Sintes-Permanyer, Victoria Ruiz de Miguel, Carlos Bravo, Judit Sacanell, María-Antonia Ramon, Laura Romero, María Deu, Antonio Román

**Affiliations:** 1 Servei de Pneumologia, Hospital Universitari Vall d’Hebron, Universitat Autònoma de Barcelona, Barcelona, Spain; 2 Ciber Enfermedades Respiratorias (Ciberes); 3 Servei de Medicina Intensiva, Hospital Universitari Vall d’Hebron, Universitat Autònoma de Barcelona, Barcelona, Spain; 4 Servei de Cirurgia Toràcica, Hospital Universitari Vall d’Hebron, Universitat Autònoma de Barcelona, Barcelona, Spain; Thomas Jefferson University, UNITED STATES

## Abstract

KL-6 is an antigen produced mainly by damaged type II pneumocytes that is involved in interstitial lung disease. Chronic lung allograft dysfunction (CLAD) after lung transplantation (LT) is a major concern for LT clinicians, especially in patients with restrictive allograft syndrome (RAS). We investigated KL-6 levels in serum and bronchoalveolar lavage fluid (BALF) as a potential biomarker of the RAS phenotype. Levels of KL-6 in serum and BALF were measured in 73 bilateral LT recipients, and patients were categorized into 4 groups: stable (ST), infection (LTI), bronchiolitis obliterans syndrome (BOS), and RAS. We also studied a healthy cohort to determine reference values for serum KL-6. The highest levels of KL-6 were found in the serum of patients with RAS (918 [487.8–1638] U/mL). No differences were found for levels of KL-6 in BALF. Using a cut-off value of 465 U/mL serum KL-6 levels was able to differentiate RAS patients from BOS patients with a sensitivity of 100% and a specificity of 75%. Furthermore, higher serum KL-6 levels were associated with a decline in Forced Vital Capacity (FVC) at 6 months after sample collection. Therefore, KL-6 in serum may well be a potential biomarker for differentiating between the BOS and RAS phenotypes of CLAD in LT recipients.

## Introduction

Chronic lung allograft dysfunction (CLAD) is one of the main challenges facing lung transplantation (LT) clinicians which continues to affect long-term survival [1]. A robust description for the term CLAD including its definition, etiology, phenotypes, pathology, treatment and outcome, has been recently published by the Pulmonary Council of the International Society for Heart and Lung Transplantation (ISHLT) [2]. In the ISHLT report CLAD was defined as a substantial and persistent decline (≥ 20%) in measured forced expiratory volume in 1 second (FEV_1_) value from the reference (baseline) value. Being the baseline computed as the mean of the best 2 post-operative FEV_1_ measurements (taken > 3 weeks apart) after exclusion of other possible causes of graft deterioration (e.g., infection, acute rejection, neutrophilic reversible allograft dysfunction) [2]. That report focused on the 3 phenotypes described to date: Bronchiolitis Obliterans Syndrome (BOS), Restrictive Allograft Syndrome (RAS) and the mixed phenotype. BOS is the most common type and is the development of airflow limitation, caused by bronchiolitis obliterans (BO). It is characterized by an obstructive pattern in the spirometry defined by a fall in FEV_1_ ≥20% from baseline and associated with other indices of airflow limitation such as FEV_1_/FVC˃0.7, (FVC, Forced Vital Capacity) without persistent radiologic pulmonary opacities [2]. RAS is a rarer phenotype accounting for approximately a quarter of CLAD cases [3]. This phenotype is characterized by a restrictive defect that should be diagnosed by a decline in FEV_1_ ≥20% (±FVC) and a ≥10% decline in total lung capacity (TLC), both relative to baselines values [2]. However, as very recently published in the RAS consensus of the ISHLT, in the absence of utilizing the TLC, a restrictive disorder can be identified from spirometry if the FVC is reduced from the baseline and the ratio of FEV_1_/FVC is elevated or increased from baseline [4]. Lung function parameters decline should be accompanied by presence of persistent opacities on chest imaging (ground glass, consolidation, small linear and reticular) that can be multilobar and/or show increasing pleural thickening consistent with a diagnosis of pulmonary and/or pleural fibrosis to diagnose RAS. In the mixed phenotype characteristics of both phenotypes mentioned are present [2].

In the ISHLT report the consensus opinions of experts in the LT field highlight the importance to clinical sub-typing into phenotypes once a probable diagnosis of CLAD has been made to stratify potential investigations and therapies, including entry into clinical trials if available [2]. Adding objective tests to better phenotype CLAD will facilitate collaboration among centers doing research on CLAD and will allow further clinical trials with specific CLAD phenotypes.

Currently, there are no specific features that can be obtained from bronchoalveolar lavage (BAL), biopsy or serum that are useful to phenotype CLAD. Although they have a major role in the detection of treatable causes prior to the diagnosis of definitive CLAD [2]. However, our group recently described a pattern of cytokines in bronchoalveolar lavage fluid (BALF) [5] and highlighted differences between CLAD subtypes. In that sense, and considering the fibrotic component of CLAD [6], it would be of interest to study biomarkers of fibrosis that could help in the diagnosis of RAS.

Krebs von den Lungen 6 (KL-6) is a circulating high-molecular-weight mucinous glycoprotein. It possesses an undefined sialylated carbohydrate chain that is recognized by a specific monoclonal antibody [7]. There is scarce information about the role of KL-6 in normal physiology. Some authors have hypothesised that is a key molecule involved in epithelial-mesenchymal interactions [8]. The epithelial-mesenchymal transition (EMT) is a response by the whole organism to generate multiple layers and complex organs during normal development, and it is also a response to pathological conditions such as tumorigenesis, hypoxia, or chronic inflammation. It is also known that in the adult organism, plastic transition between epithelial and mesenchymal cell types occurs physiology during wound healing and remodelling of tissues that develop postnatally, such as the mammary gland [9]. Thus, it can be hypothesized that KL-6 plays a role of wound healing as KL-6 is moderately expressed in type II pneumocytes and respiratory bronchiolar epithelial cells and only weakly expressed in basal cells of the terminal bronchiolar epithelium of normal lung tissues, but it is highly expressed on the cell surface of regenerating type II pneumocytes [10]. In addition, In vitro experiments have revealed a chemotactic [11], pro-proliferative and anti-apoptotic activity of KL-6 on fibroblasts, suggesting that it may play a pathological role in fibrosing lung diseases [8].

Several studies have demonstrated that KL-6 is elevated in both the BALF and the serum of patients with various types of interstitial lung diseases [12–15]. Information on KL-6 in the LT population is scarce [16–19]. In 2006, Walter et al. found that LT patients with BOS had higher levels of KL-6 [16]. However, all of the studies performed but two were performed before RAS was described, and only one of those two studies analysed RAS patients separately [17], although the results were not conclusive. Hence, the usefulness of KL-6 for CLAD phenotyping has not yet been established. We analysed levels of KL-6 in the serum and BALF of LT recipients in various clinical situations. Our main objective was to evaluate the usefulness of KL-6 as a marker to distinguish patients with BOS and RAS phenotypes easily and accurately. As a secondary objective we wanted to determine reference values of KL-6 in serum in a healthy European Mediterranean population.

## Material and methods

### 1. Study population

We performed a cross-sectional study of 73 bilateral LT patients recruited at the LT Unit of Hospital Vall d’Hebron, Barcelona, Spain. All patients had survived more than 3 months after surgery. Samples were collected from June 2014 to March 2016. The exclusion criteria were contraindication for BAL procedure, patient refusal, mechanical ventilation, and malignancy. All patients received the same immunosuppressive therapy protocol, as previously described [5]. A reference population comprising 100 healthy controls with no smoking history and no respiratory disease was also included. These patients were selected to determine the reference values of serum KL-6 in relation to gender and age in a Mediterranean population. Blood samples were collected between November 2016 and February 2017 from the blood bank “Banc de Sang i Teixits, BST”. The study was approved by the Ethics Committee of Hospital Universitari Vall d’Hebron (PR-AG_26 / 2014). All participants provided written informed consent.

### 2. Classification criteria

Patients were recruited and classified into 4 groups following the classification criteria below: Group 1 included stable patients (ST), i.e., those with BOS stage 0 at the time of routine bronchoscopy (following ISHLT/ATS/ERS guidelines to grade BOS) [20], biopsy-proven exclusion of acute rejection (if available), and no signs of infection; Group 2 included LT patients diagnosed with active infection (LTI), i.e., based on positive culture in BALF or sputum and clinical symptoms; Group 3 included patients with CLAD stratified as BOS, which was defined using the ISHLT definitions, i.e., a decline in FEV_1_ to <80% of baseline over a 3-week period and not due to other causes [20]; and Group 4 included patients with CLAD classified as RAS, which was defined as a decrease of ≥10% compared with the best post-operative TLC or ≥20% compared with the best post-operative FVC value [21]. These spirometry data had to be accompanied by a characteristic radiology pattern and/or compatible histology findings [3, 6, 22]. Pulmonary function tests were analysed using spirometry, and values were expressed as cubic centimetres or percentages of predicted normal values.

### 3. Bronchoscopy procedure and BALF sample collection

Our centre has no specific bronchoscopy surveillance protocol, although we perform at least 2 bronchoscopies during the first year: one before the patient is discharged after LT and another at any time during the first year to check bronchial sutures. The samples included in the ST group are those taken before discharge. All the bronchoscopies for the LTI, BOS, and RAS patients were performed owing to a decline in spirometry values or for symptoms of infection or respiratory symptoms. Bronchoscopy and BALF were performed as previously described [5]. Prior to sample collection, 10 ml of lidocaine 2% was administered as local anaesthetic and immediately aspirated. The tip of the bronchoscope was wedged into a sub-division of a segmental bronchus chosen depending on the suspected pathology. When no disease was suspected, the middle lobe was sampled. BAL was then performed by instillation of three separate 50-ml aliquots. The returned fractions were pooled, noting the final recovered volume, and used for several analyses including microbiological cultures. A fraction was immediately centrifuged at 1000 *g* for 10 minutes at 4ºC. Cell-free BALF supernatants were stored at –80ºC for subsequent immunoassay.

### 4. Serum sample collection

Before bronchoscopy, a peripheral venous blood sample was taken in a BD Vacutainer® Plastic SST II Advance Tube. Blood was centrifuged for 10 minutes at 1300 *g*. Serum was obtained and stored at –80°C until used.

### 5. Assessment of KL-6 levels

KL-6 was measured in serum and BALF samples using the ELISA kit for KL-6 (Eida Co. Ltd., Tokyo, Japan) according to the manufacturer’s protocol.

### 6. Statistical analysis

A descriptive analysis of the sociodemographic, clinical, and spirometric features of all the patients was conducted, and the results were stratified according to post-transplant status (ST, LTI, BOS, or RAS). Qualitative variables were expressed as absolute frequencies and percentages; quantitative variables were expressed as median (interquartile range; IQR). The association between serum and BALF KL-6 levels was studied in LT recipients and in healthy controls using the Mann-Whitney or Kruskal-Wallis test with multiple comparisons. Dunn’s correction was applied for the quantitative variables, and the chi-square or Fisher exact test was applied for the qualitative variables. A test for trends was performed using the variable of interest as an ordinal determinant in a linear regression model and KL-6 levels as a continuous outcome. Multivariate regression models were built to assess the association between serum or BALF KL-6 levels and the study groups after adjusting for time between LT and BALF sampling. The results were considered statistically significant when p<0.05. The analyses were conducted with STATA 12.0 (Stata Comp, College station, Texas, USA) and GraphPad InStat4 (GraphPad Software Inc, San Diego, California, USA).

## Results

### 1. Study population

Table 1 shows the main sociodemographic and clinical characteristics of the population studied. The procedure was bilateral in all cases. Neither acute rejection nor bronchial stenosis was observed in any patient. In total, 18 patients with LT were in stable condition (ST), 24 were infected (LTI), 20 had BOS, and 11 had RAS. There were no significant differences in age or sex between the groups. The only difference detected was for underlying disease between the ST and LTI groups. There were more patients with LTI with chronic obstructive pulmonary disease. LTI consisted of bacterial isolation in 14 patients (58%), fungal isolation in 3 patients, and coinfection of bacterial and viral infection in 7 patients. All patients had tracheobronchitis, and none had pneumonia. At the time of sample collection, the only significant difference observed between the groups was in FEV_1_ in the LTI group compared with the ST group (p = 0.044). The time from transplantation to BALF sampling was longer in recipients with BOS and RAS (p<0.001). There was no difference in elapsed time between LT and diagnosis of chronic rejection in the BOS and RAS groups or between the time from diagnosis of chronic rejection to BALF collection (p = 0.707, p = 0.340, respectively). In the case of patients with BOS, following ISHLT/ATS/ERS guidelines to grade severity of BOS [20], 3 patients presented BOS grade 0-p, 11 patients BOS grade 2, and 6 patients BOS grade 3. The characteristics of RAS patients are described in Table 2.

**Table 1:** Sociodemographic and clinical characteristics of the study population according to their post-transplant status.

Sociodemographic and clinical variables	Total n = 73	ST n = 18	LTI n = 24	BOS n = 20	RAS n = 11	p_1_	p_2_	p_3_	p_4_	p_5_	p_6_	p_7_
Age	50.7 (41.9–57.8)	52.6 (45.6–58.2)	51.8 (43.7–59.2)	49.6 (31.8–52.4)	52.2 (48.8–56.3)	0.209	0.482	0.195	0.999	0.195	0.946	0.301
Male gender	41 (56.2)	9 (50.0)	16 (66.7)	10 (50.0)	6 (54.6)	0.631	0.348	0.999	0.999	0.359	0.708	0.999
Underlying disease												
COPD	35 (47.9)	5 (27.8)	16 (66.6)	10 (50.0)	4 (36.4)	0.073	**0.028**	0.198	0.694	0.359	0.144	0.707
ILD	18 (24.7)	7 (38.9)	3 (12.5)	4 (20.0)	4 (36.4)	0.177	0.070	0.288	0.999	0.684	0.171	0.405
CF	11 (15.1)	5 (27.8)	1 (4.2)	4 (20.0)	1 (9.0)	0.149	0.068	0.709	0.362	0.160	0.536	0.631
BQ	7 (9.6)	1 (5.5)	3 (12.5)	1 (5.0)	2 (18.2)	0.532	0.623	0.999	0.539	0.614	0.640	0.281
PH	2 (2.7)	-	1 (4.2)	1 (5.0)	-	0.999	0.999	0.999	-	0.999	0.999	0.999
FVC at BALF (% pred)	64 (53–75)	67 (55–78)	63 (46–74)	69 (57–75)	59 (48–76)	0.384	0.576	0.470	0.478	0.517	0.904	0.384
FEV_1_ at BALF (% pred)	57 (46–71)	68 (57–84)	54 (38–67)	55 (48–62)	61 (37–81)	0.065	**0.044**	0.057	0.347	0.472	0.763	0.559
Time from LT to BALF (months)	22.0 (6.0–52.2)	6.0 (4.1–17.1)	12.3 (3.9–30.2)	67.0 (33.8–92.3)	35.9 (19.8–47.4)	<0.001	0.159	**<0.001**	**0.006**	**<0.001**	**0.038**	0.233
Time from LT to diagnosis of CLAD (months)	43.8 (34.1–70.6)	-	-	48.0 (31.2–84.3)	40.7 (34.1–48.3)	-	-	-	-	-	-	0.707
Time from CLAD to BALF (months)	1.01 (0.4–7.7)	-	-	2.3 (0.2–13.9)	0.7 (0.5–1.9)	-	-	-	-	-	-	0.340

Data are presented as n (%) or median (p25-p75). COPD, chronic obstructive pulmonary disease; ILD, interstitial lung disease; CF, cystic fibrosis; BQ, bronchiectasis; PH, Pulmonary Hypertension; FVC, force vital capacity; FEV_1_, forced expiratory volume in one-second; BALF, Bronchoalveolar lavage fluid; pred, Predicted; LT, Lung Transplant; CLAD, Chronic Lung Allograft Dysfunction; ST, stable patients; LTI, Lung Transplant patient with active Infection; BOS, bronchiolitis syndrome; RAS, Restrictive Allograft Dysfunction; p1: p-value of comparison between all groups; p2: p-value of ST vs. LTI; p3: p-value of ST vs. BOS, p4: p-value of ST vs. RAS, p5: p-value of LTI vs. BOS; p6: p-value of LTI vs. RAS; p7: p-value of BOS vs. RAS.

**Table 2:** Main features of RAS population.

	Age	Underlying disease	KL-6 serum (U/mL)	Pathology	Radiology	Time to RAS diagnosis (months)	Survival	Time from RAS diagnosis to death (months)
1	22	CF	1455.8	Interstitial inflammation	Opacities	26	Dead	6
2	52	COPD	1638	NA	Opacities Upper/lower lobes	48	Alive	
3	63	ILD	3178	NA	Opacities Upper lobes	20	Alive	
4	48	BQ	483.8	NA	Opacities Upper lobes	16	Alive	
5	54	ILD	1171.03	Organizing pneumonia	Ground glass	5	Dead	8
6	53	COPD	4165.8	Organizing Pneumonia	Ground glass	20	Dead	11
7	32	BQ	487.75	NA	“white lung”	9	Dead	2
8	54	ILD	918	NA	Opacities	13	Alive	
9	56	ILD	465.35	Interstitial pneumonia	Opacities Upper lobes	64	Alive	
10	65	COPD	556.5	NA	Opacities	19	Alive	
11	50	COPD	913.85	Interstitial pneumonia	Opacities Lower lobes	17	Dead	33

CF, cystic fibrosis; COPD, chronic obstructive pulmonary disease; ILD, interstitial lung disease; BQ, bronchiectasis; NA, not available.

### 2. Analysis of serum KL-6 levels

#### 2.1. Healthy controls–reference values

**Fig 1:**
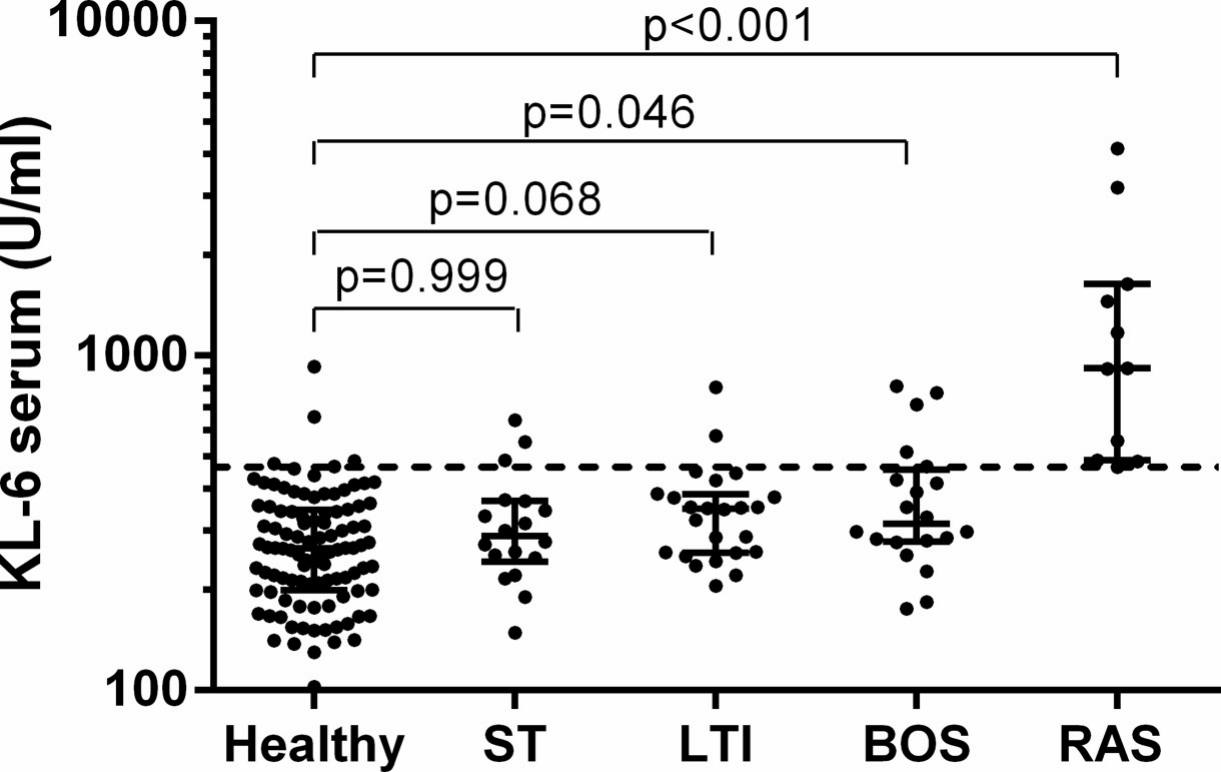
Comparison of serum KL-6 levels between healthy controls and lung transplant recipients. Solid lines indicate median and interquartile range. Dashed line indicates the cut-off value of 465U/ml. ST, Stable patients; LTI, Lung Transplant patients with an active Infection; BOS, Bronchiolitis Obliterans Syndrome; RAS; Restrictive Allograft Syndrome.

The influence of gender and age on KL-6 levels was evaluated in healthy controls. No differences were found regarding KL-6 levels according to gender; however, levels did tend to increase with age (Table 3). No significant differences were observed regarding KL-6 levels between healthy controls, ST or LTI recipients (Fig 1).

**Table 3:** Description of serum KL-6 levels in healthy controls according to sex and age groups.

KL-6 serum (U/ml)	KL-6 serum (U/mL)	P-value
Overall (n = 100)	265.8 (199.4–345.4)	-
Sex		0.278
Male (n = 50)	273.2 (207.8–386.3)	
Females (n = 50)	253.8 (198.3–315.9)	
Age		0.035*
20–30 (n = 20)	220.6 (166.7–311.5)	
31–40 (n = 20)	228.0 (197.9–296.0)	
41–50 (n = 20)	287.9 (228.8–362.5)	
51–60 (n = 20)	265.8 (194.7–377.2)	
61–65 (n = 20)	315.6 (247.0–390.2)	

*p-value for trend.

#### 2.2. Cohort of LT patients

**Fig 2:**
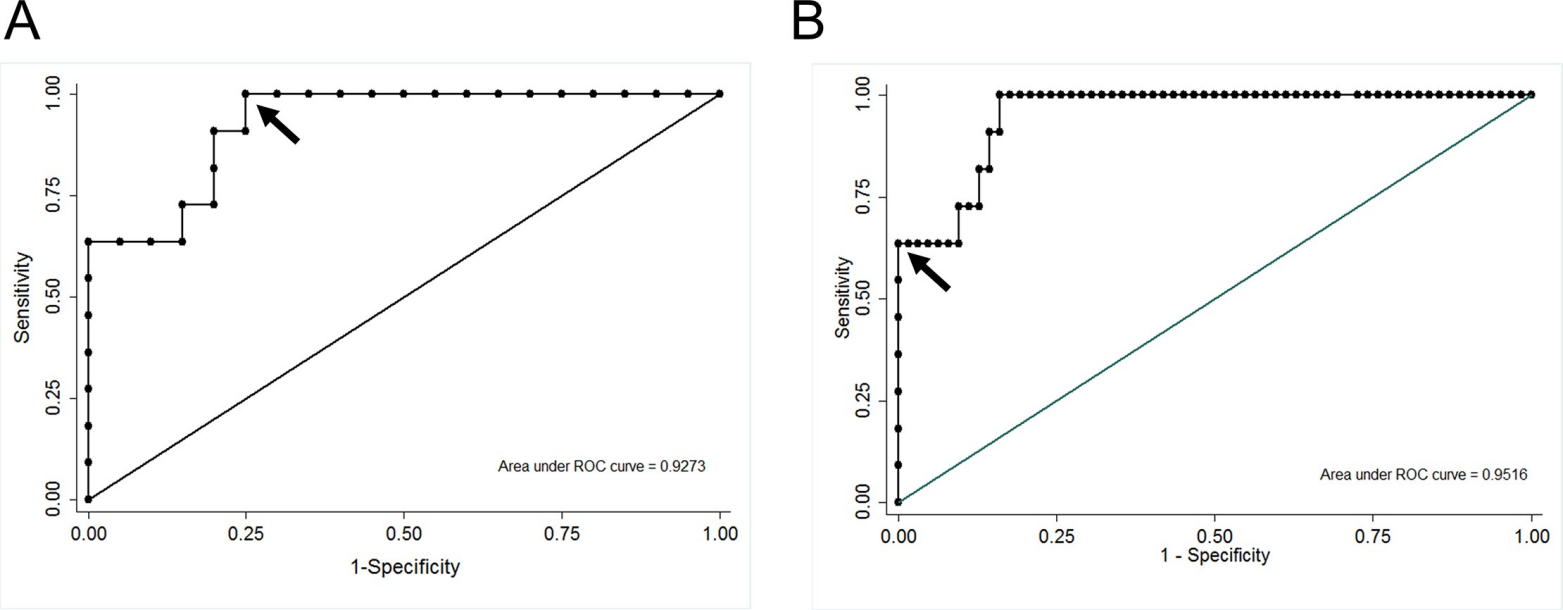
**Receiver operating curve (ROC) analysis: A)** To discriminate between RAS and BOS by KL-6 serum levels. The arrow indicates the cut-off value of 465 U/mL which gives a 100% sensitivity and 75% specificity for the test with a positive predictive value of 69% and a negative predictive value of 100%. **B)** To discriminate between RAS and the whole LT population. The arrow indicates the cut-off value of 800 U/mL which gives a 64% sensitivity and 97% specificity for the test with a positive predictive value of 78% and a negative predictive value of 94%.

Serum levels of KL-6 are shown by group in Fig 1. The highest levels were observed in the RAS group, with a median (IQR) level of 918 (487.8–1638) U/mL. Serum KL-6 levels in RAS patients were significantly higher than in any of the other groups (p<0.001) (Table 4). Those differences were significant even after adjustment for time elapsed between lung transplantation and BALF collection (Table 4). The area under the curve for serum KL-6 levels to discriminate between RAS and BOS patients was 0.93. Setting a cut-off value of 465 U/mL, the test had 100% sensitivity, 75% specificity, a 69% positive predictive value (PPV) and a 100% negative predictive value (NPV) (Fig 2A). The area under the curve to discriminate RAS patients from the whole LT population was 0.95. To achieve a good PPV and NPV, when comparing with the whole population, the cut-off value was set at 800 U/mL obtaining a sensitivity of 64%, specificity of 97%, PPV of 78%, and NPV of 94% (Fig 2B). No correlation between serum KL-6 levels and demographic characteristics was observed.

**Table 4:** Serum and BALF KL-6 levels according to post-transplant status.

	Total* n = 73	ST n = 18	LTI n = 24§	BOS n = 20	RAS n = 11§	p_1_	p_2_	p_3_	p_4_	p_5_	p_6_	p_7_
Serum KL-6 level (U/mL)	350.1 (259.5–483.4)	289.1 (248.8–367.8)	348.5 (257.9–382.5)	313.8 (278.5–446.3)	918.0 (487.8–1638.0)	**<0.001**	0.999	0.836	**<0.001** 0.001*	0.999	**<0.001** <0.001*	**<0.001** <0.001*
BALF KL-6 level	143.1 (88.4–249.6)	153.7 (103.8–283.8)	120.0 (48.6–157.2)	186.9 (95.2–269.5)	234.1 (111.1–535.2)	0.065	0.199	0.999	0.999	0.144	0.062	0.999
BALF KL-6 level corrected for proteins	1.9 (0 .8–3.0)	2.0 (0.7–4.9)	1.1 (0.4–2.1)	2.4 (1.4–3.7)	1.8 (0.7–2.4)	**0.041**	0.208	0.999	0.999	**0.015** 0.002*	0.637	0.960

Data are presented as median (p25-p75). BALF, Bronchoalveolar lavage fluid; ST, stable patients; LTI, Lung Transplant patient with active Infection; BOS, bronchiolitis syndrome; RAS, Restrictive Allograft Dysfunction; p1: p-value of comparison between all groups; p2: p-value of ST vs. LTI; p3: p-value of ST vs. BOS, p4: p-value of ST vs. RAS, p5: p-value of LTI vs. BOS; p6: p-value of LTI vs. RAS; p7: p-value of BOS vs. RAS.

* p-value after adjustment for time elapsed between lung transplant and BALF.

§ Some variables have missing values: one in the BALF KL-6 level and one in the BALF KL-6 level corrected for proteins.

### 3. Analysis of KL-6 in BALF

The values of KL-6 in BALF are presented in Table 4. No differences were observed between the groups. Same results were obtained when BALF KL-6 levels were normalized by total protein content in BALF in order to take into account alveolar-capillary barrier conditions [23]. There was no correlation between serum KL-6 levels and BALF (p = 0.71).

### 4. Analysis of lung function and correlation with serum KL-6 levels

**Fig 3:**
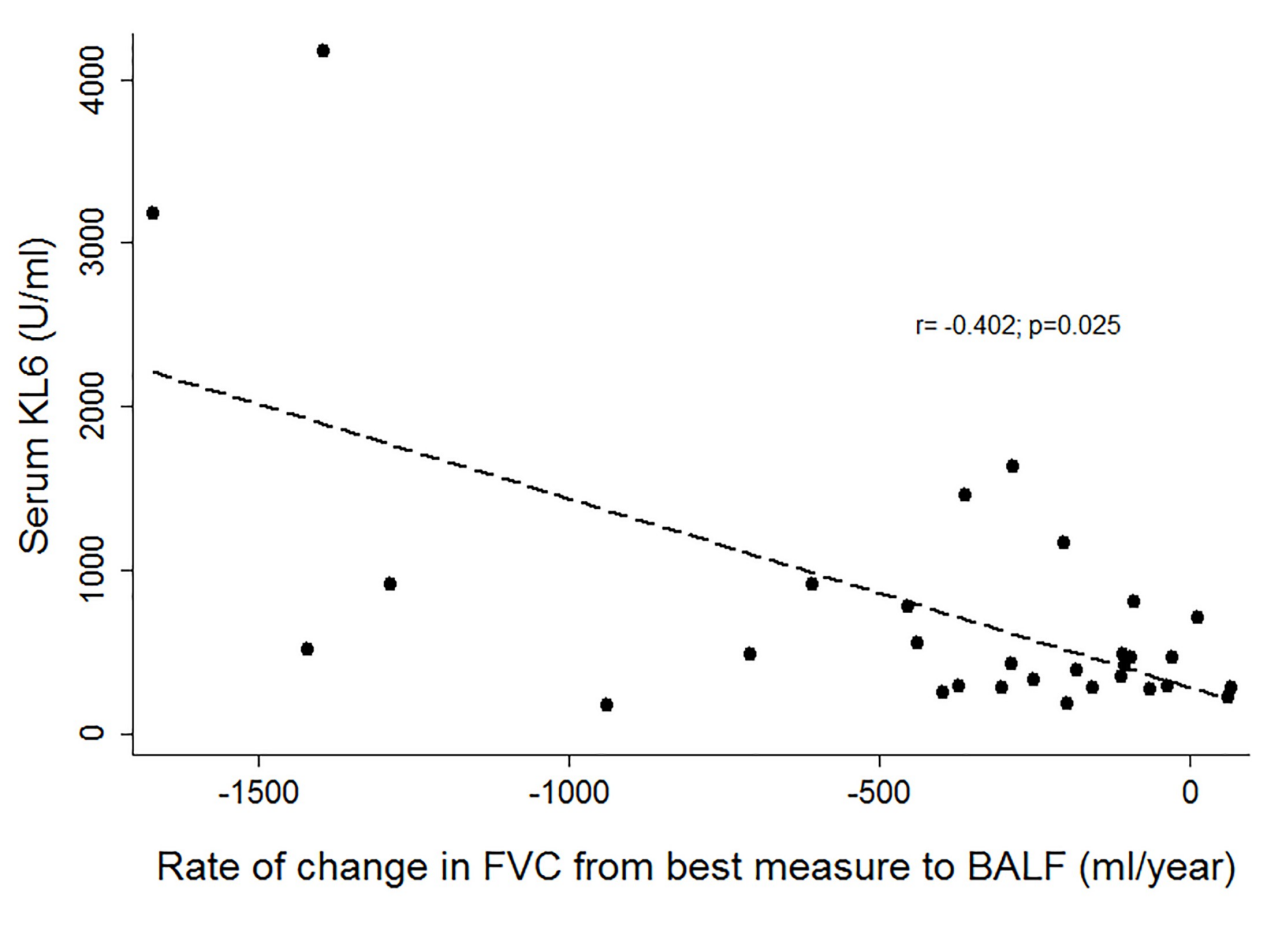
Correlation between the rate of change per year in forced vital capacity (FVC) from baseline to enrollment and serum KL-6 levels in CLAD patients.

In CLAD patients (BOS and RAS) the Spearman correlation coefficient showed no association between the change rate of FEV_1_ from baseline (best FEV_1_ after LT) to FEV_1_ at sample collection and serum KL-6 levels. However, there was a significant correlation between the rate of change in FVC from baseline to enrollment and KL-6 levels (r = –0.402, p = 0.025) (Fig 3).

**Fig 4:**
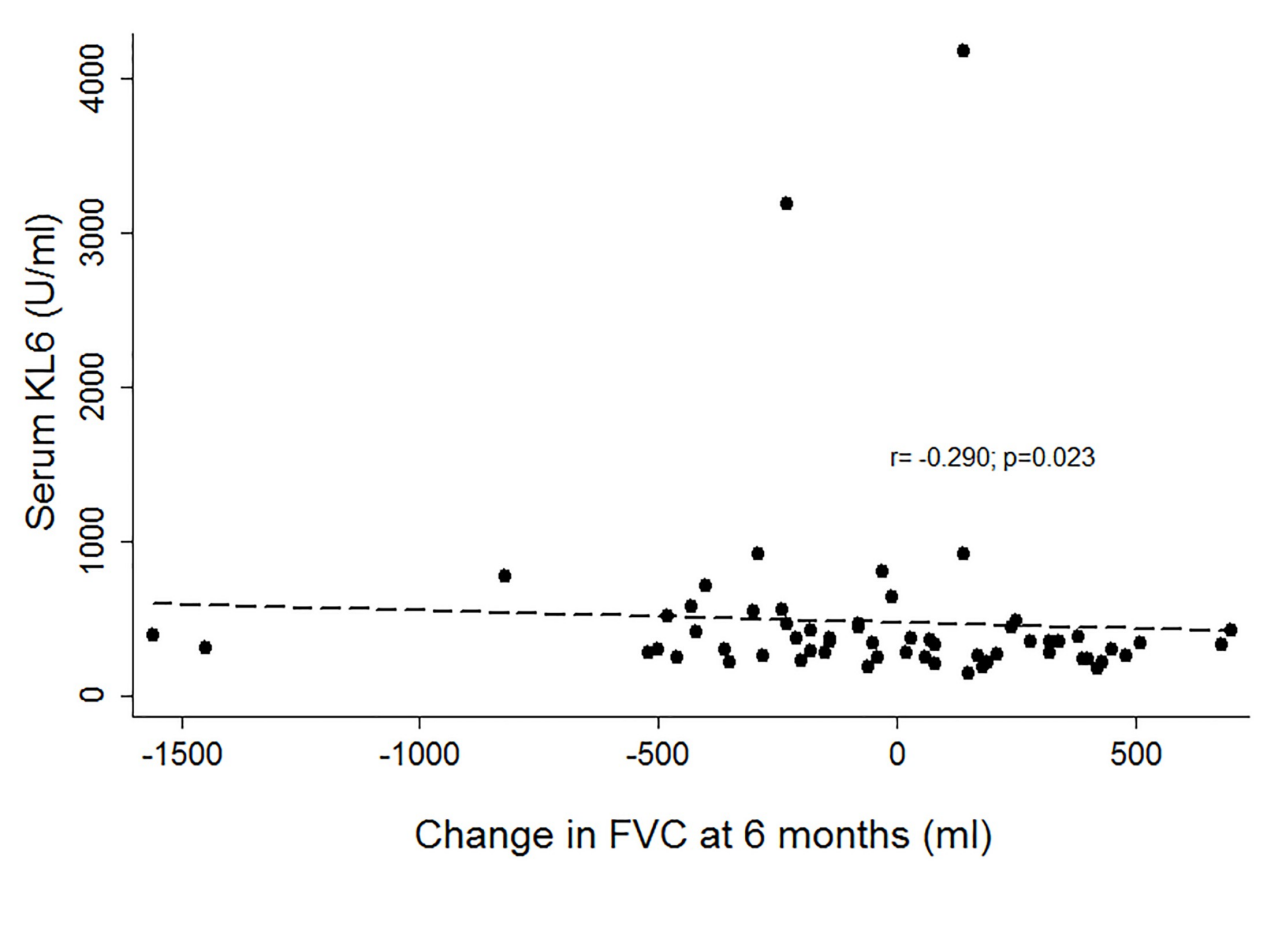
Correlation between rate of change from forced vital capacity (FVC) at the time of sample collection to FVC at 6 months after sample collection and serum KL-6 levels.

Lung function was also evaluated after 3 and 6 months from sample collection. The Spearman correlation coefficient was used to study the relationship between serum KL-6 levels and drop of FEV_1_ and FVC after sampling. There was no correlation between serum KL-6 levels and change of FEV_1_ from the day of sample collection to 3 or 6 months after. The only significant correlation was observed between KL-6 levels and drop of FVC from sampling to 6 months (r = –0.29; p = 0.023) (Fig 4).

## Discussion

The discovery of diagnostic biomarkers for RAS have potential utility in clinical decision-making as well as in pharmaceutical research. To the best of our knowledge, this is the first study to investigate levels of KL-6 as a tool for phenotyping CLAD patients. We found that bilateral LT recipients with RAS had higher levels of KL-6 in serum than the other groups (healthy controls, ST, LTI, and BOS). In contrast, the absence of higher KL-6 levels in BALF samples suggests that BALF samples are not suitable for determining KL-6 with the aim of detecting RAS. Also novel in our study is the establishment of reference KL-6 values in healthy controls in Spain.

One of the goals of the study was to determine normal values of KL-6 in a healthy European Mediterranean population, since it has previously been reported that polymorphisms identified in the *MUC1* gene of German and Japanese patients affect serum KL-6 levels [24]. We also assessed the impact of age and gender on these values. KL-6 values were not impacted by gender, although there was a trend towards increased values with age. Therefore, reference values should be staged by age. Normal values in our healthy Mediterranean population were similar to those found in other healthy populations [15–17]. We also found that KL-6 levels in the serum of healthy controls were similar to those observed in stable LT recipients, as reported elsewhere [16, 17], suggesting that fibrogenesis is scarcely active as a mechanism of lung repair in stable LT patients.

Effective pharmacologic therapy of CLAD remains an unmet medical need. Retransplantation may be the only therapeutic option for advanced CLAD for well-selected patients, but with worse results when compare with first LT especially in the case of RAS [2]. An earlier detection of RAS could guide the clinician to perform a closer follow up of those patients. RAS patients could also benefit from being included in clinical trials of new anti-fibrotic treatments that have shown their efficacy in other fibrotic diseases. The future should be focus on multicentre collaborative studies to design clinical trials specific for each CLAD phenotype in order to improve LT outcome [2]. To do so, it will be crucial to perform an accurate phenotyping of CLAD patients with the help of objective biomarkers such as KL-6. The characteristics of the RAS phenotype, a marked proliferation of fibroblasts in various areas of the pulmonary interstitium and the resulting disease severity [25], support the hypothesis that expression and activity of markers of lung injury and fibrosis are elevated in this phenotype. Thus, the analysis of a marker such as KL-6, which promotes proliferation and survival of lung fibroblasts [8], was thought to be a reliable option for differentiation between those phenotypes. Up to date, few studies have analysed KL-6 levels in LT recipients and all of them had small sample sizes and only serum samples were analysed [16–19]. Furthermore, most of them were performed before the description of the RAS phenotype, consequently, at the time, the CLAD phenotypes were not differentiated.

In our study, BOS patients had higher serum KL-6 levels than healthy controls; however, we did not find significant differences between BOS patients and stable LT patients. Moreover, we found the highest KL-6 values in patients with RAS. No correlation was found between serum KL-6 levels and loss of FEV_1_, although we did observe a correlation with change in FVC from baseline when CLAD patients were analysed. Those results seem to be in disagreement with those found in 2006 by Walter et al. [16] who found higher serum KL-6 levels in patients with BOS and observed a significant correlation between serum levels of KL-6 and a decrease in FEV_1_ from the post-transplant baseline to the time of enrollment. However, it is important to bear in mind that the results of both studies are difficult to compare, as Walter et al. published their findings before the description of the RAS phenotype.

For the first time we suggested a cut-off value for KL-6 levels in serum to phenotype CLAD patients. We proposed a threshold value of 465 U/mL to discriminate between BOS and RAS patients using the ELISA method (see above) in order to ensure a very high NPV (100%). Therefore, all RAS patients would be detected, albeit with a fairly low false-positive rate (69% PPV). When using this threshold value to discriminate RAS from the whole LT population, the NPV remained at 100%, but the PPV dropped to 52%. Consequently, a cut-off of 800 U/mL was proposed in order to discriminate the RAS population from the LT population, with a PPV of 78% and an NPV of 94%. Earlier studies assess cut-off values for KL-6 levels to predict BOS. In 2010 Haberman et al. in their longitudinal study suggested that change in KL-6 level above baseline level of >200 U/mL was a more specific predictor of BOS than levels of KL-6 at a specific time point [18]. Additionally, in a very recent study, Bessa et al. also analysed the change in KL-6 over time at two defined time points in 21 LT patients, of whom only 9 developed BOS and none RAS [19]. The authors determined that a decrease of 11% in KL-6 levels over time was the best cut-off value for development of BOS. However, they did not find significant differences in serum KL-6 levels during follow-up between patients with and without BOS. The change in KL-6 levels could not be evaluated in our study owing to its cross-sectional design. Thus, absolute KL-6 levels were assessed.

To our knowledge the only study were serum KL-6 was analysed in RAS patients was published in 2011 by Ohshimo et al. [17], albeit as a secondary objective. In that study, results from the first part are hard to compare with those obtained in the second part, as in the initial portion, BOS patients were defined following the ISHLT definition from 2002 [26]. In the second part the authors redefined BOS patients following the new terminology for CLAD by classifying patients as obstructive CLAD and RAS [3]. In the first part, KL-6 levels for BOS patients were 611 ± 500 U/mL, whereas in the second analysis, in which patients were classified as obstructive CLAD, levels of KL-6 were strikingly lower (361 ± 292 U/mL). Thus, heterogeneity in definitions makes it difficult to compare results from the first part of the study with our results. The results from the second part of the study are in line with our results, as the authors also observed that the highest levels of KL-6 were in the RAS group. Furthermore, the range of levels in the RAS group, 851 ± 519 U/mL, was very similar to that found in our study. One of the novelties of our study is that we established a cut-off point for KL-6 levels in serum that allowed us to discriminate between both CLAD phenotypes (BOS and RAS) in serum, with a high sensitivity, specificity, PPV, and NPV.

To our knowledge, this is the first study to measure BALF KL-6 levels in an LT population. Levels of KL-6 in BALF were lower than serum levels, and there was no correlation between the levels obtained between the 2 kinds of samples. Moreover, BALF KL-6 levels could not discriminate between LT groups. In contrast, a correlation between KL-6 levels in both kinds of samples was observed by Nathani et al. [27] in patients with acute respiratory distress syndrome. However, given the type of patient, the authors obtained samples very early after the injury. Kohno et al. [15] studying healthy individuals, patients with idiopathic pulmonary fibrosis, sarcoidosis or hypersensitivity pneumonitis found a correlation between KL-6 levels in BALF and serum and also found higher values of KL-6 in serum samples. Similarly, in their study of chronic beryllium disease, Inoue et al. [28] found that serum KL-6 proved superior to BALF KL-6 for discriminating between beryllium-sensitized patients and healthy controls [28].

As suggested by Inoue et al. [28], serum KL-6 is more sensitive than BALF KL-6 for several reasons. The first involves the dilutional effect of lavage fluid relative to serum: epithelial lining fluid contains higher levels of KL-6 than BALF and serum samples. Second, both the destruction of the alveolar-capillary barrier and the enhancement of alveolar capillary permeability are thought to be necessary for the leakage of KL-6 into systemic circulation, since KL-6 is high-molecular-weight glycoprotein [10]. Thus, this could explain why some time after the insult, we can detect higher KL-6 levels in peripheral blood. The cross-sectional design of our study prevented us from seeing early stages of development of RAS, and we probably only observed severe alveolar epithelial damage. This could be the reason why serum KL-6 proved to be a better indicator in our study. Furthermore, it has been suggested that increased serum KL-6 levels do not reflect the intensity of inflammation, but rather indicate the extent of damaged alveolar epithelium and the alveolar capillary permeability that appears after multiple insults [10].

In summary, we provide relevant data on normal serum KL-6 values in a healthy European Mediterranean population and show that KL-6 serum levels could help to differentiate between RAS and BOS lung recipients. However, this study has several limitations. One limitation of this study is that sample size was relatively small, in particular in the RAS group. A validation study with an independent and larger cohort of patients it is needed to corroborate the potential diagnostic value of KL-6 as a RAS phenotype biomarker. Furthermore, classification of patients has been done by current definitions, but not all patients have TLC values available and FVC decline has been used as a surrogate of restriction. Finally, this study was cross-sectional and serum KL-6 levels were not measured before the onset of CLAD. Thus, future investigations should include longitudinal studies to determine the predictive value of KL-6 in serum for earlier diagnosis of RAS and the prognostic value of the biomarker in terms of survival. Future studies might explore whether KL-6 levels could help in the monitoring of future CLAD treatments. In addition, the differences in KL-6 levels found between RAS and BOS could highlight a differential pathophysiological mechanism in both clinical entities that should be further studied to better understand and treat those phenotypes.

In conclusion, serum KL-6 level seems to be a useful biomarker for differentiating between the BOS and RAS phenotypes of CLAD. However, further studies are needed to confirm the usefulness of this biomarker in clinical practice and its predictive ability.

## Supporting information

S1 Table Metadata in a cross-sectional lung transplanted cohort to study KL-6 levels. (XLS)
